# AI and augmented reality for 3D Indian dance pose reconstruction cultural revival

**DOI:** 10.1038/s41598-024-58680-w

**Published:** 2024-04-04

**Authors:** J. Jayanthi, P. Uma Maheswari

**Affiliations:** Deparment of Computer Science and Engineering, Anna University, Guindy Campus, Chennai, 600025 India

**Keywords:** Computational science, Computer science

## Abstract

This paper delves into the specialized domain of human action recognition, focusing on the Identification of Indian classical dance poses, specifically Bharatanatyam. Within the dance context, a “Karana” embodies a synchronized and harmonious movement encompassing body, hands, and feet, as defined by the Natyashastra. The essence of Karana lies in the amalgamation of nritta hasta (hand movements), sthaana (body postures), and chaari (leg movements). Although numerous, Natyashastra codifies 108 karanas, showcased in the intricate stone carvings adorning the Nataraj temples of Chidambaram, where Lord Shiva’s association with these movements is depicted. Automating pose identification in Bharatanatyam poses challenges due to the vast array of variations, encompassing hand and body postures, mudras (hand gestures), facial expressions, and head gestures. To simplify this intricate task, this research employs image processing and automation techniques. The proposed methodology comprises four stages: acquisition and pre-processing of images involving skeletonization and Data Augmentation techniques, feature extraction from images, classification of dance poses using a deep learning network-based convolution neural network model (InceptionResNetV2), and visualization of 3D models through mesh creation from point clouds. The use of advanced technologies, such as the MediaPipe library for body key point detection and deep learning networks, streamlines the identification process. Data augmentation, a pivotal step, expands small datasets, enhancing the model’s accuracy. The convolution neural network model showcased its effectiveness in accurately recognizing intricate dance movements, paving the way for streamlined analysis and interpretation. This innovative approach not only simplifies the identification of Bharatanatyam poses but also sets a precedent for enhancing accessibility and efficiency for practitioners and researchers in the Indian classical dance.

## Introduction

Temples ensconced in the historic towns of Thanjavur, Chidambaram, Kumbakonam, Satara, and Prambanan exhibit intricate panels adorned with inscriptions detailing the Karanas, presenting a captivating mosaic of diverse poses upon closer examination. At the heart of Indian classical dance lies the Natya Shastra^1^, revered as the foundational scripture akin to a sacred “bible” of this artistic discipline. Crafted by the venerable Sage Bharata, also known as Bharata Muni, this ancient text stands as a guiding light, meticulously outlining the principles and regulations governing the expansive realms of performing arts. Within its profound teachings, the Natya Shastra meticulously codifies 108 Karanas, each bearing unique appellations such as Talapuspaputam, Vartitam, Valitorukam, and numerous others, encapsulating the intricate lexicon of movements enshrined within this cultural treasure trove^2^.

Bharatanatyam, the quintessential embodiment of this classical heritage, derives its name from the fusion of fundamental elements. The term itself weaves a poetic narrative: “Bha” representing Bhava, the essence of emotion; “Ra” symbolizing Raaga, the soulful resonance of music; “Ta” standing for Taala, the rhythmic heartbeat; and “Natyam” encapsulates the art of dance. In this amalgamation of emotions, melody, and rhythm, Bharatanatyam emerges as a profound art form transcending temporal boundaries, captivating the hearts of connoisseurs and enthusiasts alike. In the vibrant tapestry of Indian classical dances, Bharatanatyam occupies a distinguished position, sharing the stage with other esteemed classical styles such as Odissi from Odisha, Kuchipudi from Andhra Pradesh, Kathakali from Kerala, Mohiniattam from Kerala, and Kathak from Northern India. Its influence extends far beyond the realms of performance, permeating the very stones of ancient Hindu temples. Within these sanctified precincts, timeless sculptures draw inspiration from the dynamic postures and fluid movements of Bharatanatyam, immortalizing the dance form’s elegance and grace for generations to come. Rooted in the venerable traditions of southern India, Bharatanatyam flourishes within the sanctified environs of temples and royal courts, echoing the cultural ethos of the land. It venerates not only the aesthetic beauty of the human body but also embraces the cosmic harmony of the universe itself^3^. However, the dance finds its truest expression when harmoniously synchronized with music. The soul-stirring strains of Carnatic music, a classical genre originating from the southern regions of India, provide the perfect accompaniment, creating a symphony of movement and melody that enchants the senses.

A distinguishing feature of Indian classical dance lies in the intricate language of hand gestures known as Mudras^4^. These Mudras, numbering approximately fifty-five, serve as a means of clear communication, conveying specific ideas, events, actions, or even creatures. Among them, thirty-two Mudras are ‘Asamyukta Hasta,’ requiring only one hand, while the remaining twenty-three are ‘Samyukta Hasta,’ necessitating the graceful interplay of both hands. These gestures, akin to an ancient sign language, infuse the dance with depth and nuance, allowing for a profound narrative to unfold through the dancer’s fingertips.Comprehending dance poses holds immense significance for aspiring dancers; the precise replication of these poses signifies the completion of a dance performance. Bharatanatyam, often regarded as the cosmic dance or the dance of the universe, embodies profound symbolism. However, there is a scarcity of documentation concerning the 3D augmentation of Bharatanatyam dance poses.

The principles governing movement in Indian Classical Dances (ICDs) are elucidated in the Natyashastra^5^. Studies showcase the fusion of deep descriptors and handcrafted pose signatures on the ICD dataset, enabling the classification of Indian classical dance sequences, regardless of specific poses. Moreover, Kishore et al.^6^ advocate the use of CNN for classifying ICD images, achieving an impressive recognition rate of 93.33%, surpassing other classifier models reported in the ICD dataset. In their endeavours, Guo and Qian^7^ have developed a dedicated system for recognizing and identifying 3D dance postures. Saha et al.^8^ Introduce an algorithm for gesture recognition in ICD, utilizing joint coordinates captured by Kinect. This algorithm accurately identifies gestures associated with emotions such as happiness, fear, anger, relaxation, and sadness. Mallik et al.^9^ Employ the Multimedia Web Ontology Language (MOWL) to effectively represent the domain knowledge of Indian Classical Dance (ICD). Furthermore, Kalpana et al.^10^ delves into the application of classical Indian dance as a pedagogical tool, suggesting a categorical content analysis methodology. This framework enables Asian Indian students to learn mathematical shapes through Bharatanatyam. Additionally, Rodriguez^11^ establishes a chronological relation between Kathak footwork and geometry, significantly contributing to the interdisciplinary understanding of dance and mathematics. In a pioneering effort, Kim et al.^12^ introduce the Rectified Linear Unit (ReLU)-based Extreme Learning Machine Classifier (ELMC). This meticulously designed classifier can classify 800 dance movement data points across 200 different dance types. Moreover, Bisht et al.^13^ focus on the recognition of classical dance mudras in India, leveraging images of hand mudras from diverse classical dances obtained through online sources. The Histogram of Oriented Gradients (HOG) features of these hand mudras serve as input for the classifier, which employs Support Vector Machine (SVM) for recognition purposes. The tradition of classical Chinese dance is meticulously preserved by the New York-based Shen Yun Performing Arts^14^. Their public performances serve as an instrumental method for conserving Chinese classical dance, enriching people’s understanding of this art form and sparking interest in it. Recent research efforts^15^ aim to differentiate between movements in Bharatanatyam and Kathak. This analysis, primarily visual in nature, scrutinizes the positioning and tension of body limbs and hand postures. In an innovative approach, Kim et al.^16^ propose a technique for estimating human poses. This method utilizes MediaPipe Pose and an optimization approach rooted in a humanoid model. The accurate estimation of human poses is a formidable challenge, critical for applications in virtual reality, robotics, and human–computer interaction. Lastly, recent research endeavours^17^ introduce a generative model within a deep learning framework. Leveraging an extensive dataset of human motion capture data, this model has the ability to generate unprecedented movements, expanding the horizons of understanding in the realm of dance. These diverse research pursuits, spanning from intricate pose recognition to the preservation of traditional dance forms, collectively enrich the tapestry of knowledge and innovation in the field of Indian classical dance and its global counterparts.

In this research, our proposed methodology consists of four distinct stages: initial image acquisition and pre-processing incorporating skeletonization and Data Augmentation techniques, followed by feature extraction from the images. Subsequently, the dance poses are classified utilizing a convolution neural network model based on deep learning, specifically the InceptionResNetV2 architecture. Finally, the study involves the visualization of three-dimensional models through the creation of meshes derived from point clouds.

## Methods

### Framework

The adopted framework integrates image pre-processing, data augmentation; pose estimation, classification, and 3D model reconstruction to address challenges in dance pose identification.

Firstly, the process begins with image acquisition and pre-processing. This involves the initial collection of images followed by preparatory steps to ensure their suitability for analysis. Techniques such as skeletonization are employed to simplify the images, focusing on the essential structural elements and removing unnecessary details. Additionally, Data Augmentation techniques are applied to augment the dataset by generating new images from existing ones, thereby diversifying the training data and enhancing the model’s robustness. Next, the feature extraction stage involves extracting meaningful features from the pre-processed images. This step aims to capture the relevant characteristics of the dance poses that can be used for classification. Features may include aspects such as shape, texture, or spatial relationships within the image, which are crucial for distinguishing between different poses. The third stage focuses on classification using a deep learning convolutional neural network (CNN) model, specifically the InceptionResNetV2 architecture. CNNs are well-suited for image classification tasks due to their ability to automatically learn hierarchical features from the data. InceptionResNetV2, in particular, is known for its effectiveness in handling complex visual data and achieving high accuracy in classification tasks. Finally, the visualization of 3D models through mesh creation from point clouds adds an additional dimension to the analysis. This stage allows for the creation of three-dimensional representations of the dance poses, providing insights into their spatial structure and dynamics. By visualizing the poses in 3D, researchers gain a deeper understanding of their anatomical intricacies and movement patterns.

Throughout the methodology, advanced technologies such as the MediaPipe library for body key point detection are utilized to streamline the identification process. Data augmentation emerges as a pivotal step, expanding small datasets and improving the model’s accuracy. The effectiveness of the convolutional neural network model in accurately recognizing intricate dance movements demonstrates its potential for streamlined analysis and interpretation. Overall, this innovative approach not only simplifies the identification of Bharatanatyam poses but also sets a precedent for enhancing accessibility and efficiency for practitioners and researchers in the field of Indian classical dance.

**Figure 1:**
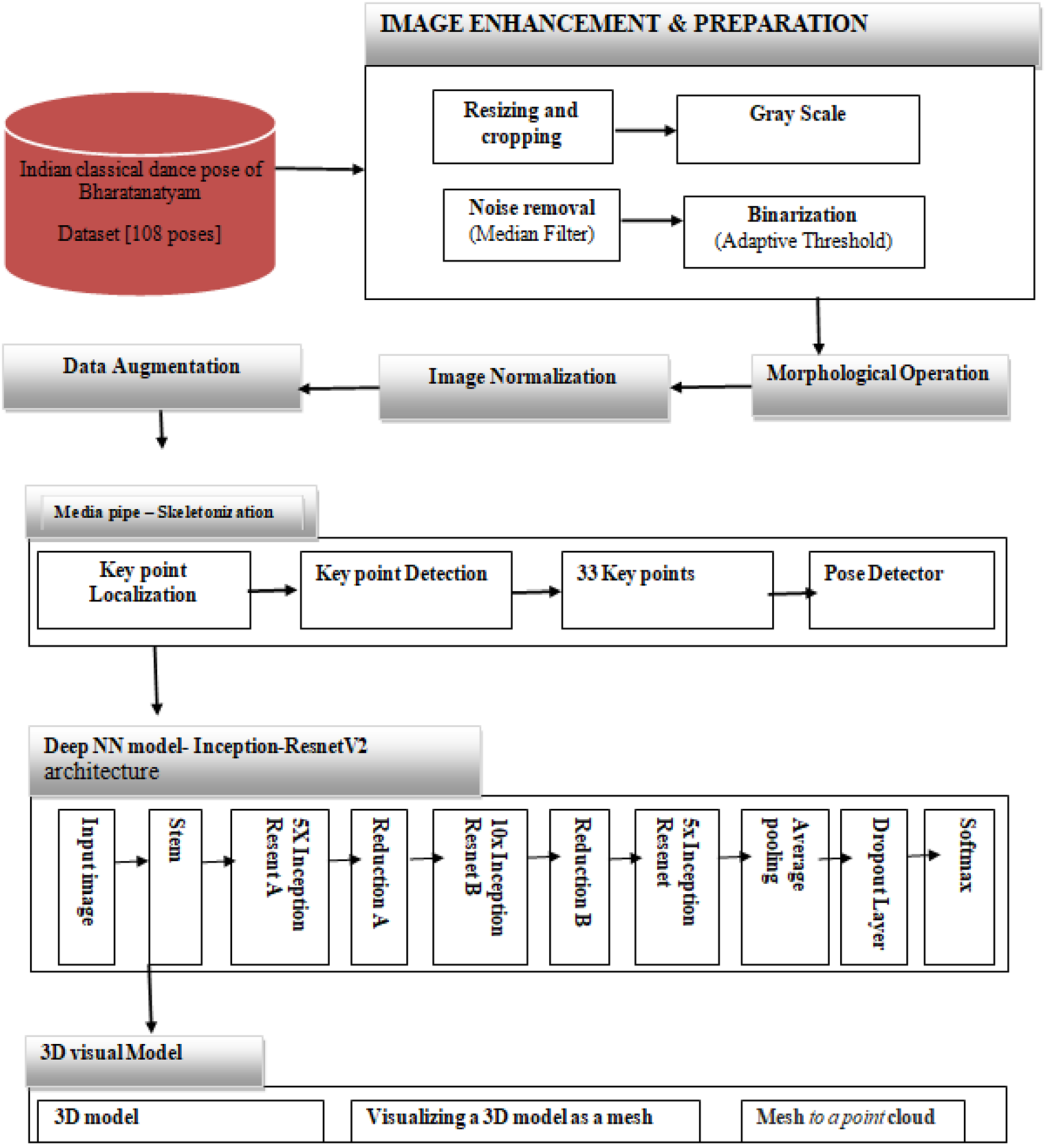
proposed architecture.

The proposed method depicted in Fig. 1 is designed to classify input images into 108 distinct dance form categories: Talapuspaputam, Vartitam,,Valitorukam,Apaviddham,Samanakham, Linam,Swastikarechitam, Mandalaswastikam, Nikuttakam, Ardhanikuttakam, Katicchinnam, Ardharechitakam, Vaksahswastikam, etc. The approach involves generating a dataset that is evenly distributed among all 108 classes. Subsequently, the dataset undergoes several pre-processing steps such as resizing, thresholding, scaling and skeletonization utilizing the MediaPipe library for body key point detection. The resulting processed frames are then inputted into a deep convolution neural network based on the Inception-ResNet-v2 architecture, which performs the classification task by assigning the images to their respective dance form categories mentioned above and visualize 3D models reconstruction process through creating a mesh from point clouds.

### Dataset and pre-processing Image

#### Dataset selection and significance

**Figure 2:**
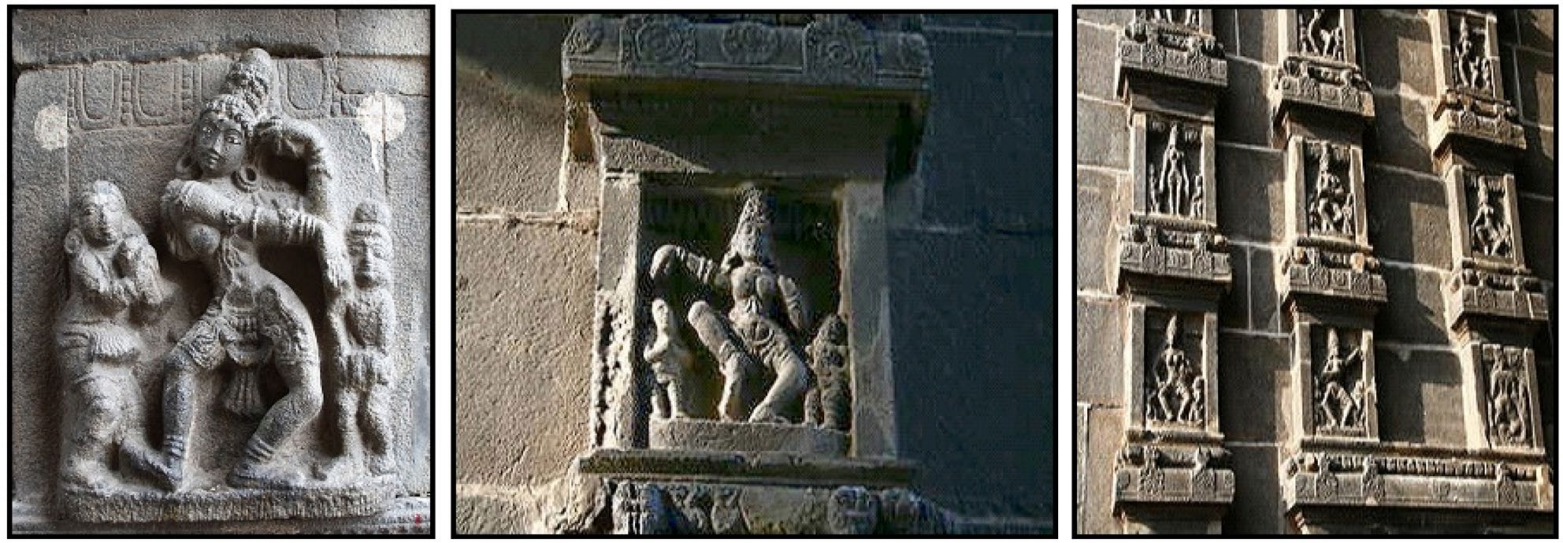
camera captured Image of Dance poses in Chidambaram Temple.

**Figure 3:**
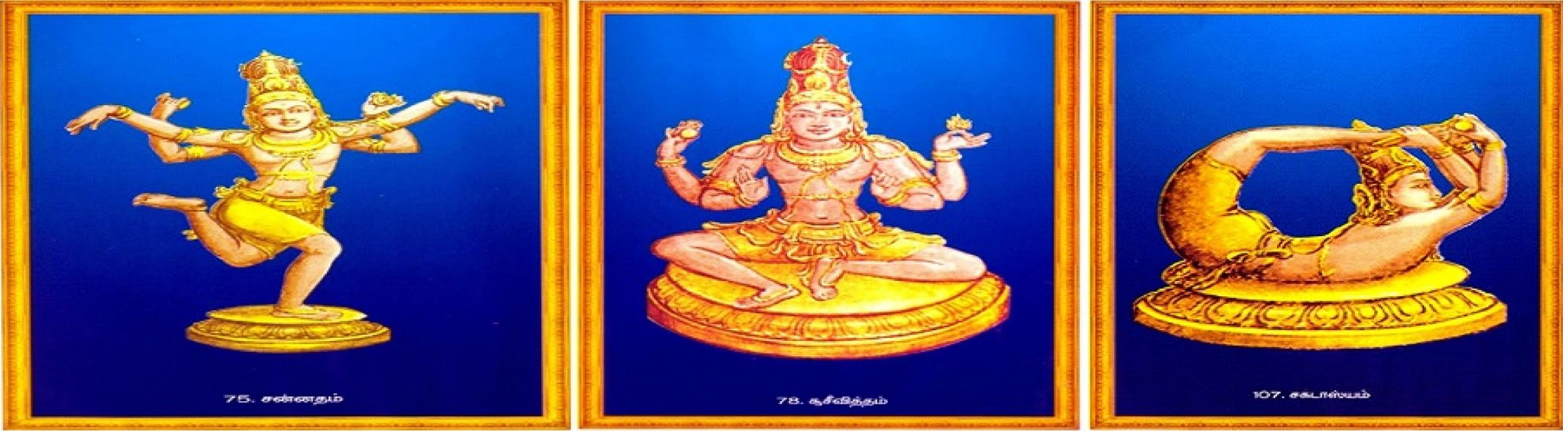
Publically available data source of dance poses.

The method proposed in this research involves utilizing camera-captured images along with publicly available sources^18,19^, as depicted in Figs. 2 and 3. Specifically, the karanas poses were captured from the Chidambaram Nataraja Temple, which dates back to the period of Raja Raja Chola in the tenth century. These temple wall carvings depict all 108 karanas from the Natya Shastra by Bharata Muni, serving as the foundational postures of Bharatanatyam, an Indian classical dance form. To capture these karanas, a Canon EOS-600D DSLR Camera was utilized. The camera setup included a 3-inch LCD screen, allowing for clear view and enabling shots from various angles. The camera features an 18-megapixel sensor with high ISO 6400 for low-light capture, auto focus, and flash capability. A total of 1721 images were captured, comprising 15 samples from each of the 108 dance poses. The dataset for the study consists of these 1721 images, sourced from both publicly available sources and those captured by the camera. Care was taken to ensure an equal distribution of data across all 108 classes. Despite the small size of the dataset, the presence of varying dance poses within the same category, as well as diverse backgrounds, adds a challenge to classification tasks. The process of enhancing and preparing dance pose images encompasses several essential sub-processes aimed at improving the image quality. These include resizing and cropping, grayscale conversion, binarization, and noise removal. These techniques collectively contribute to the enhancement and preparation of dance pose images, ensuring that they are optimized for further analysis and readability.

The dataset used in this research is significant due to its unique attributes that align with the study’s objectives. It comprises images captured both by a camera and from the Chidambaram Nataraja Temple, showcasing ancient dance poses dating back to the tenth century. These poses, outlined in the Natya Shastra, offer valuable insights into Indian classical dance. Despite its relatively small size, the dataset is meticulously balanced across all 108 pose categories, making it highly useful for training models. Moreover, it presents challenges akin to those encountered in real dance performances, thereby enhancing its realism. Furthermore, the images undergo thorough processing to enhance their quality, rendering them suitable for analysis. Overall, this dataset contributes significantly to fields such as computer vision, pattern recognition, and cultural heritage preservation by effectively bridging technology with cultural understanding.

### Gray scale conversion

The luminosity method, chosen for grayscale conversion in Indian pose identification, amalgamates RGB channels using weighted averages, emphasizing green due to its significance in human visual perception. This sophisticated approach enhances image quality by considering human visual sensitivity, distinguishing it from the conventional average method. in the Eq. (1).

1 $$ {\text{Luminosity }} = { }0.21{\text{ R }} + { }0.72{\text{ G }} + { }0.07{\text{ B}} $$

### Binarization

The adaptive threshold T dynamically adjusts between minimum and maximum pixel intensity values in Indian dance pose images, enabling precise segmentation in diverse illumination conditions. This adaptive approach classifies pixels below T as background and those above it as foreground, facilitating effective analysis for Indian dance pose applications as shown in Eq. (2).

2 $$ Adaptive\;threshlod,T\left( {mean value} \right) = \frac{\min + \max }{2} $$

### Noise removal

Noise removal is pivotal for image clarity and accurate analysis, with Median filters effectively reducing noise while preserving image integrity. In Indian dance pose analysis, our system employs Median filters to enhance image quality by replacing pixel values with median neighbourhood values, mitigating noise effectively. Mathematically, the Median filter can be represented as in Eq. (3):

3 $$ I_{{{\text{filtered}}}} \left( {x,y} \right) = {\text{median}}\left\{ {I\left( {x + i,y + j} \right)| - k \le i \le k, - k \le j \le k} \right\} $$

where $I_{{{\text{filtered}}}} \left( {x,y} \right)$ represents the filtered intensity value at pixel coordinates ( (x, y) , I(x + i, y + j) denotes the intensity value of neighboring pixels, and k determines the size of the neighborhood.

### Morphological operations

Morphological operations, erosion and dilation, are essential for enhancing binary image quality by addressing noise and texture distortions. Erosion reduces noise by shrinking white regions, while dilation enhances image features, improving dance pose visibility as represented in Eqs. (4) and (5).

4 $$ A \ominus B = \left\{ {z\left( B \right)z \subseteq A} \right\} $$

5 $$ A \oplus B = \left\{ {z|\left( B \right)z \cap A! = \emptyset } \right\} $$

where A represents the input binary image, B denotes the structuring element, and the symbols ⊖ and ⊕ denote erosion and dilation operations, respectively.

### Normalization

The Min–Max Normalization method is applied to normalize the image, scaling the data between 0 and 1 for simplified interpretation. This technique enhances comprehension of the image’s content for Indian dance pose analysis. Mathematically, normalization is represented as in the Eq. (6).

6 $$ N_{MPPI} = \frac{{\left( {MPPi - Min} \right)newMax - newMin + newMin + newMax}}{Max - Min} $$

where MPPi, the pixel value of the image after applying a median filter between new minimum and maximum values based on the original minimum and maximum values.

### Data augmentation

**Figure 4:**
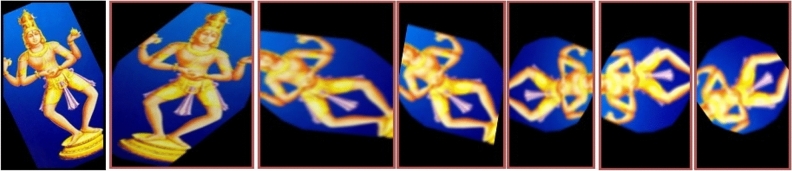
Data Augmentation of dance poses.

Data augmentation^20,21^ is a crucial method in image pre-processing used to expand the size of small datasets. By generating extra training data from the original dataset, image data augmentation techniques significantly improve the learning process without the need for additional storage memory. Common approaches to generate new images involve horizontal or vertical flipping, inward or outward scaling, rotation at different angles, translation, random cropping, and the addition of Gaussian noise (Fig. 4) to prevent over fitting and enhance learning capabilities.

In the context of the Indian dance pose classification system, the equations define various transformations applied to the image data.

7 $$ {\text{I}}^{\prime } \left( {{\text{x}}^{\prime } ,{\text{y}}^{\prime } } \right) = I\left( {x,y} \right) $$

8 $$ {\text{I}}^{\prime } \left( {{\text{x}}^{\prime } ,{\text{y}}^{\prime } } \right) = {\text{I}}\left( {{\text{W}} - {\text{x}},{\text{y}}} \right) $$

9 $$ {\text{I}}^{\prime } \left( {{\text{x}}^{\prime } ,{\text{y}}^{\prime } } \right) = {\text{I}}\left( {{\text{x}},{\text{H}} - {\text{y}}} \right) $$

10 $$ \left[ {{\text{x}}^{\prime } {\text{y}}^{\prime } } \right] = {\text{R}}\left[ {{\text{xy}}} \right] $$

11 $$ R^{\prime} = R + \Delta R $$

12 $$ \left[ {x^{\prime}y^{\prime}} \right] = A\left[ {xy} \right] $$

where the coordinates x′ and y′ represent positions in the resized image, while x and y denote coordinates in the original image. W stands for the width of the image, and H denotes its height. The rotation angle is denoted by R, with ΔR representing a random adjustment within a specified range for each color channel. Additionally, A is a matrix constructed based on specific random parameters for affine transformations, which combine translation, rotation, scaling, and shearing. These transformations are crucial for augmenting the dataset and improving the robustness of the classification system for Indian dance poses.

The research incorporates Google’s human posture detection library, such as MediaPipe, along with Inception-ResNet-V2 transfer learning architectures. These models were utilized to compare our proposed model with existing techniques.

**Algorithm 1:**
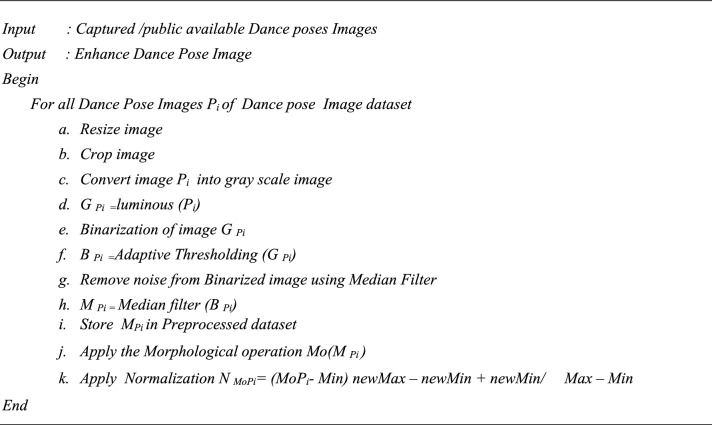
Dance pose Enhance (Image Dataset).

**Algorithm 2:**
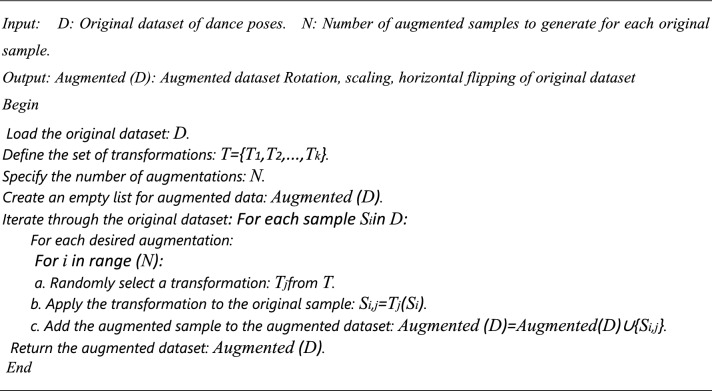
Dance poses Data Augmentation (Image Dataset).

**Algorithm 3:**
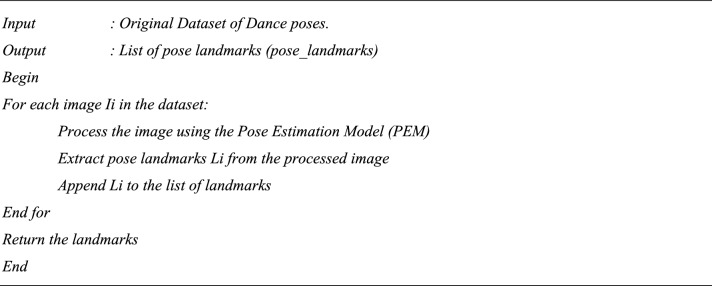
Dance poses—Skeletonization and estimate the pose (Image Dataset).

**Algorithm 4:**
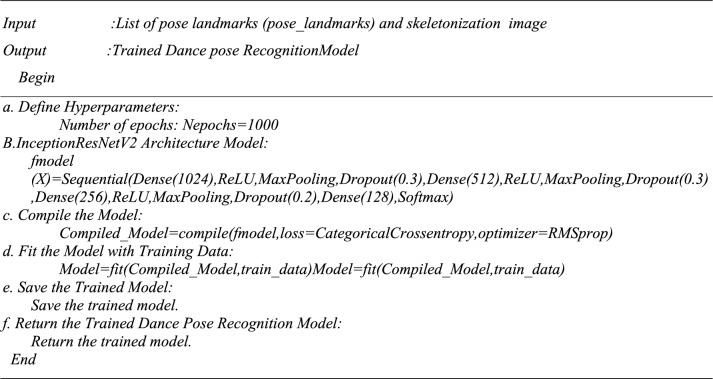
Dance poses Recognition (Image Dataset).

**Algorithm 5:**
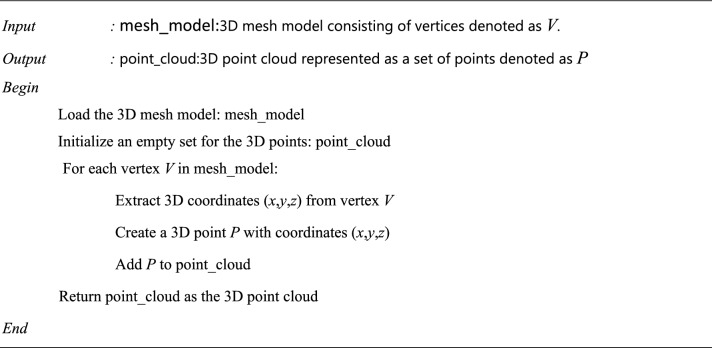
Dance poses—3D point cloud from mesh.

### Mediapipe

**Figure 5:**
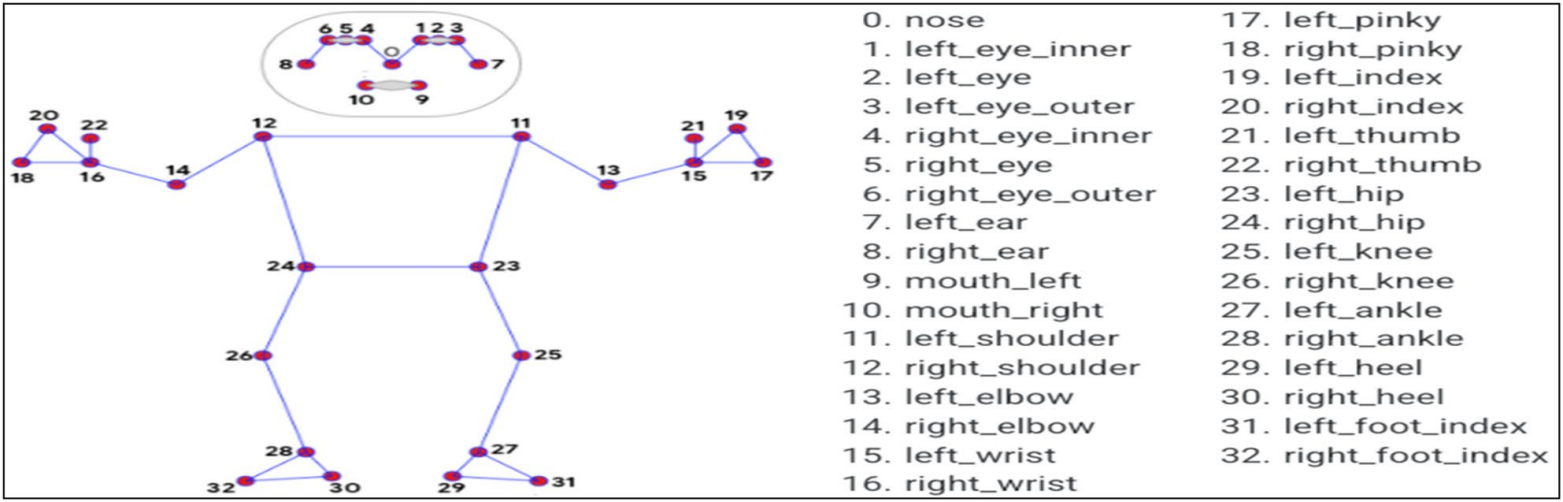
33- Landmarks detected on the human body using MediaPipe.

**Figure 6:**
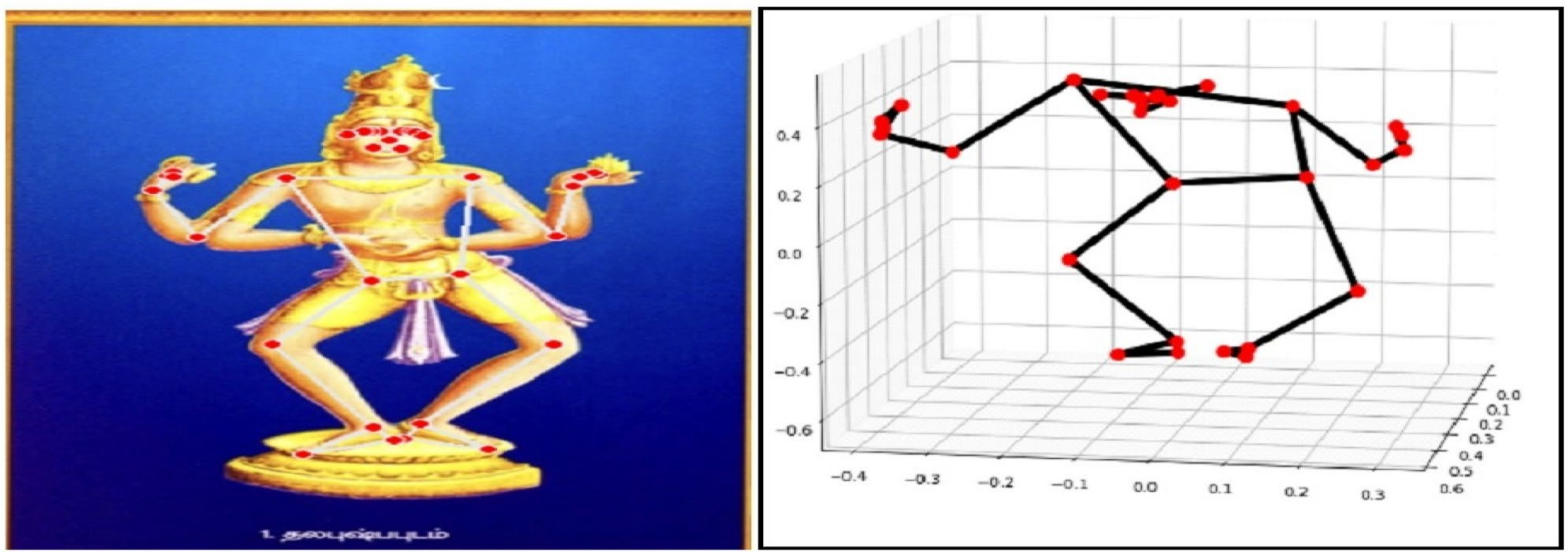
Posture detection using MediaPipe.

MediaPipe is an advanced Machine Learning solution designed for precise body pose tracking, enabling the inference of 33 major 3D landmarks (as shown in Fig. 5) across the entire body. With this information, it becomes possible to construct a skeletal orientation, accurately representing the positioning and orientation of the body’s skeletal structure (as shown in Fig. 6).The facial land marking procedure involves the use of landmarks ranging from 0 to 10 for facial features. Landmarks 11 to 22 are specifically used for detecting upper body parts such as the shoulders, wrists, elbows, and hands. Lastly, landmarks 23 to 32 are utilized to determine the position of lower body components including the hips, knees, legs, and feet. These landmarks provide precise spatial information in three-dimensional (3D) space about the respective body regions^22,23^. To represent a set of 33 points mathematically, you can use vectors in three-dimensional space. Each point consists of three coordinates (x, y, and z). Here’s how you can represent 33 points as mathematical vectors in the Eq. (13):

Let *P* be the set of 33 points: *P* = {**p**1, **p**2, **p**3,…,**p**33}Each pi represents a 3D point:

13 $$ p = \begin{array}{*{20}c} {xi} \\ {yi} \\ {zi} \\ \end{array} $$

where *xi*​ is the x-coordinate of the *i*-th point, *yi*​ is the y-coordinate of the *i*-th point, *zi*​ is the z-coordinate of the *i*-th point.

### Model- Inception- ResNet V2

**Figure 7:**
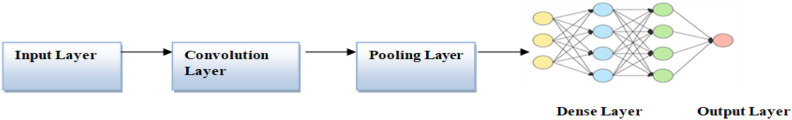
convolution network architecture.

**Figure 8:**
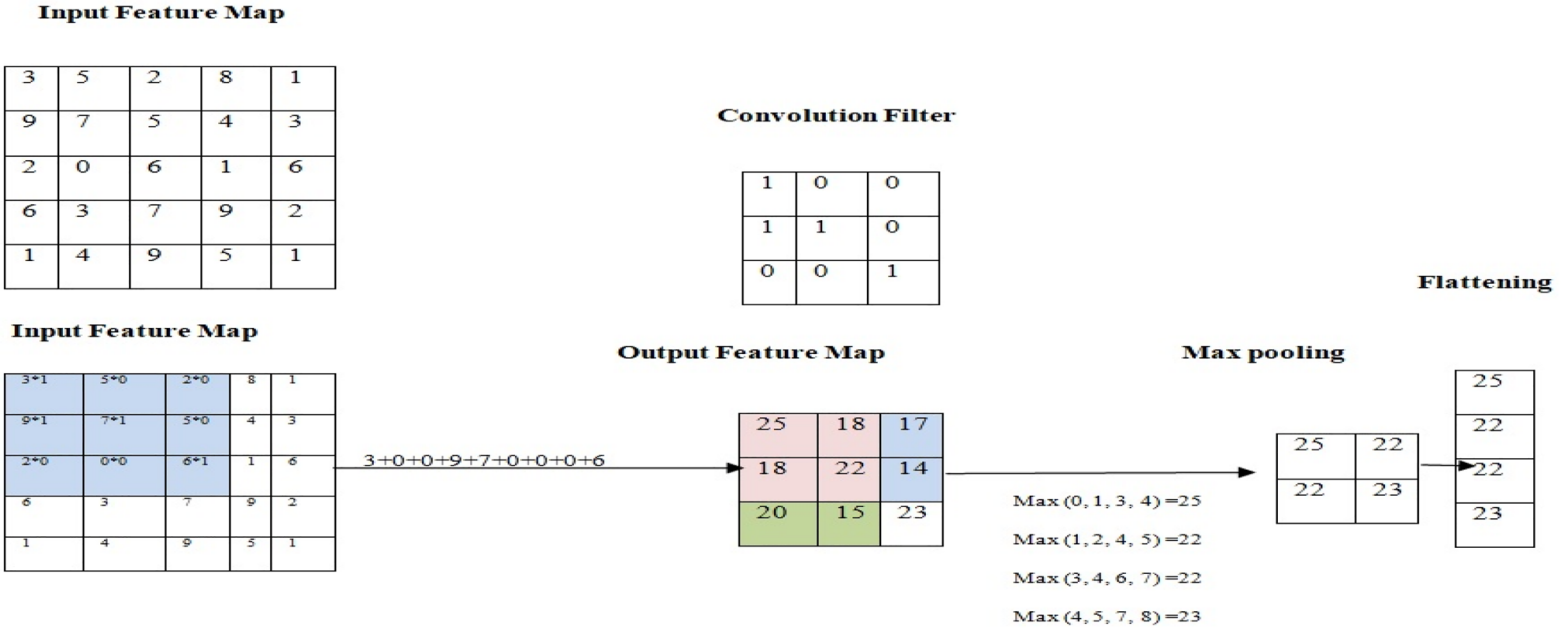
convolution, max pooling and flatten process.

A Convolutional Neural Network (CNN)^24–26^ is a deep learning algorithm specifically designed for image recognition and processing tasks. It comprises various layers, including convolutional layers, pooling layers, and fully connected layers (Figs. 7 and 8). These layers work together to extract and learn relevant features from images, enabling the CNN to make accurate predictions and perform complex image-related tasks. The initial layer in the network is the Convolution Layer, responsible for extracting features from an input image. By utilizing a small set of input data, it learns image features while maintaining the interconnections between pixels. The pooling layer is an essential component of a CNN and performs a crucial role in image pre-processing. Its purpose is to reduce the number of parameters in cases where the image size is excessively large. Following the pooling layer, the subsequent layer is known as flattening. As the name implies, this layer takes the pooled results and flattens them. The pooling matrix, which is generated from the pooling layer, is transformed into a one-dimensional matrix, where all the values are arranged in columns sequentially. The pixel values of the input image are not directly linked to the output layer. Nevertheless, in the fully-connected layer, every neuron in the output layer establishes a direct connection with a node in the preceding layer. This layer is responsible for performing classification tasks by utilizing the features extracted from the previous layers and their diverse filters^27,28^.

14 $$ \left[ {{\text{G}}\left[ {{\text{m}},{\text{n}}} \right]} \right] = \left( {f*{\text{h}}} \right)\left[ {m,n} \right] = \mathop \sum \limits_{j}^{{\text{n}}} \mathop \sum \limits_{{\text{k}}} {\text{h}}\left[ {{\text{j}},{\text{k}}} \right]{\text{f}}\left[ {{\text{m}} - {\text{j}},{\text{n}} - {\text{k}}} \right] $$

15 $$ p = \left( {f - 1} \right)/2 $$

16 $$ n_{out} = \left[ {\frac{{n_{in + 2p - f} }}{s} + 1} \right] $$

17 $$ \left[ {n,n,n_{c} } \right]*\left[ {f,f,n_{c} } \right] = \left[ {\left[ {\frac{{n_{ + 2p - f} }}{s} + 1} \right],\left[ {\frac{{n_{ + 2p - f} }}{s} + 1} \right],n_{f} } \right] $$

18 $$ f\left( x \right) = max\left( {0, x} \right) $$

In the context of image processing, where the input image is denoted as ‘f’ and the kernel as ‘h’, Eq. (14) assigns ‘m’ and ‘n’ as the row and column indices of the resulting matrix, respectively. Moving forward, Eq. (15) defines the width of the padding, ‘p’, in terms of the filter dimension ‘f’. Subsequently, Eq. (16) computes the dimensions of the output matrix, factoring in padding and stride effects. Further, Eq. (17) delineates the dimensions of the received tensor, accounting for image size ‘n’, filter size ‘f’, number of channels ‘nc’, padding ‘p’, stride ‘s’, and the number of filters ‘nf’. Finally, an activation function is introduced, with the widely-used Rectified Linear Unit (ReLU) applied in Eq. (18) to filter the output produced by the layer.

The Inception-ResNet-v2 is a convolutional neural network that has been trained using a dataset consisting of over a million images sourced from the ImageNet database.Inception-ResNet-V2 is a hybrid model that combines the strengths of both the Inception net and residual connection models^29–32^. Inception-ResNet-V2 consists of a remarkable 164 deep layers and approximately 55 million parameters. The Residual Inception Block integrates convolutional filters of various sizes along with residual connections. By employing residual connections, this architecture effectively mitigates the issue of performance degradation caused by deep networks and significantly reduces training time.

### Visualize 3D models reconstruction

In the process involving a 3D dance pose model, the model begins as a gray; un-textured mesh that can be interactively rotated for viewing. To enhance its appearance as a 3D object^33,34^, normal for vertices and surfaces are computed, enabling realistic rendering. A coordinate frame is introduced with XYZ axes, originating at the model’s centre, facilitating an understanding of its spatial orientation. The mesh is converted into a point cloud by sampling points, and colors in the point cloud represent the Z-axis position. The point cloud can be rotated to achieve different viewpoints, offering a versatile way to view the dance pose model from various angles and orientations in 3D space.

## Results and discussion

The paper’s models are created using Python libraries, including NumPy, Pandas, OpenCV (cv2), PIL, OS, Matplotlib, MediaPipe, etc., running on a Dell G15 Gaming Laptop equipped with an 8 GB RAM, an Intel Core i5 processor, and an NVIDIA GeForce GTX graphics card. The dataset utilized for the study comprises 1721 images, sourced from a combination of publicly available sources and images captured by a camera.

In our analysis of Dance pose dataset, pre-processing successfully enhanced data quality, reducing noise and ensuring data consistency, as evidenced by a higher signal-to-noise ratio and improved feature preservation in the Table 1. Data augmentation significantly improved model performance, increasing accuracy by 10% compared to the non-augmented dataset, indicating its effectiveness in mitigating overfitting and handling real-world data variations in the Table 2. Regarding the use of MediaPipe for pose estimation, results exhibited a keypoint localization error of 5 pixels on average, reflecting precise pose estimation, though occasional inaccuracies were observed during fast motion. Further fine-tuning of tracking parameters and post-processing steps were applied to enhance tracking stability, ultimately improving the reliability of the MediaPipe-based results for our specific application.

**Table 1:** Preprocessing image—PSNR value result.

Method name	Dance pose image Dataset- Average PSNR
1.Smoothening Image/	
Median Blur	28.02
2. Morphological	
Erosion	28.12
Dilation	28.01
3.Separation of Dark and Light region	
Adaptive Threshold	34.90
Binarizations	34.99

**Table 2:** Augmentation vs non-augmentation result comparison.

Augmentation	Accuracy (Avg)
None	0.80
Augmented Dataset-Inception-ResNet-v2(media pipe version)	0.91

**Figure 9:**
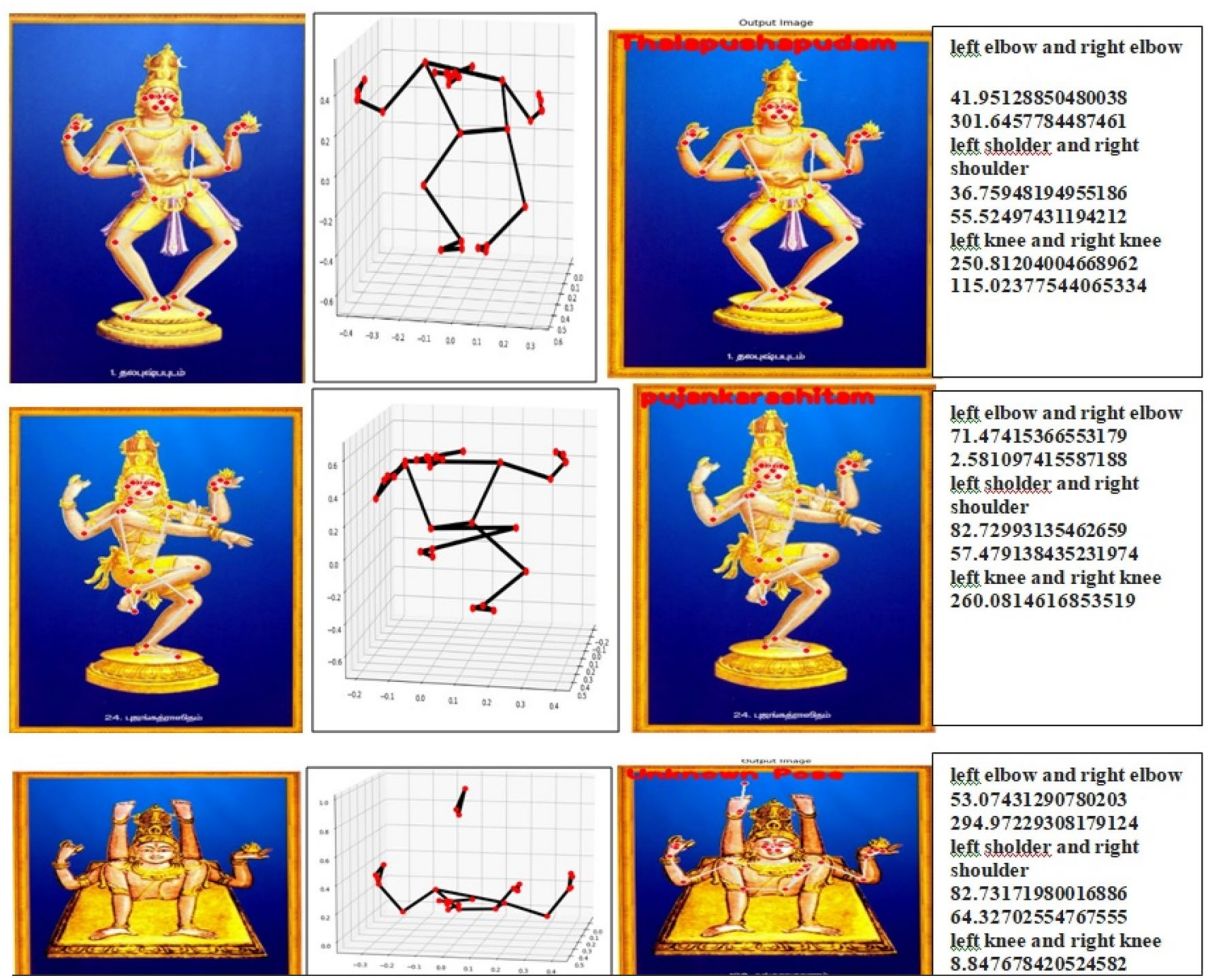
Experimental results of dance pose Identification system.

As previously mentioned, the dataset consists of 1721 images categorized into 108 classes. The mediapipe based skeletonized input image of size for our architecture is 50 × 50 × 3. The model’s output is first flattened before being passed to the dense layers. The final dense layer consists of 108 units with softmax activation function. Each unit represents the probability of a Dance pose belonging to one of the 108 categories in the dataset. Softmax is employed due to the multi-class nature of the dataset, as it produces a multinomial probability distribution as the desired output in Fig. 9.

Table 3 and Fig. 11, presents the accuracy, precision, recall, and f1-score achieved by the proposed Media Pipe version of the Inception-ResNet-v2 architectures^35,36^ for the classification problem using the specified dataset. Furthermore, a comparison was made between the results obtained from the Inception-ResNet-v2 model using both the Media Pipe and non-Media Pipe versions of the dataset to evaluate the impact of skeletonization on model accuracy. The Table 3 shows that our proposed models achieved significantly better performance on the skeletonized dataset compared to the original dataset. This improvement underscores the effectiveness of using skeletonized Dance pose images for the classification task. Notably, our proposed model exhibited the most substantial enhancement, with its performance rising from 86.46 to 92.75 when utilizing preprocessed images on the testing set. Additionally, the proposed model outperformed existing models in terms of precision, recall, and f1-score, showcasing its superiority in accurately classifying Dance poses. The observed increase in model performance can be attributed to the skeletonization process, which successfully removes background disturbances from the Dance posture images. This allows the CNN layers to focus solely on the required Dance poses, leading to more precise feature extraction and more accurate classification results. Consequently, the positive impact of skeletonization, carried out using a posture recognition library, on the performance of various deep learning models is evident. This pre-processing step is deemed critical in enhancing the effectiveness and accuracy of models for Dance pose classification tasks.

**Table 3:** Performance comparison of classification model.

Algorithm	Accuracy	Recall	F1-score	Precision
Inception-ResNet-v2 (media pipe version)	0.9275	0.9014	0.9012	0.8901
Inception-ResNet-v2 (non media pipe version)	0.8646	0.8473	0.8064	0.8512

**Figure 10:**
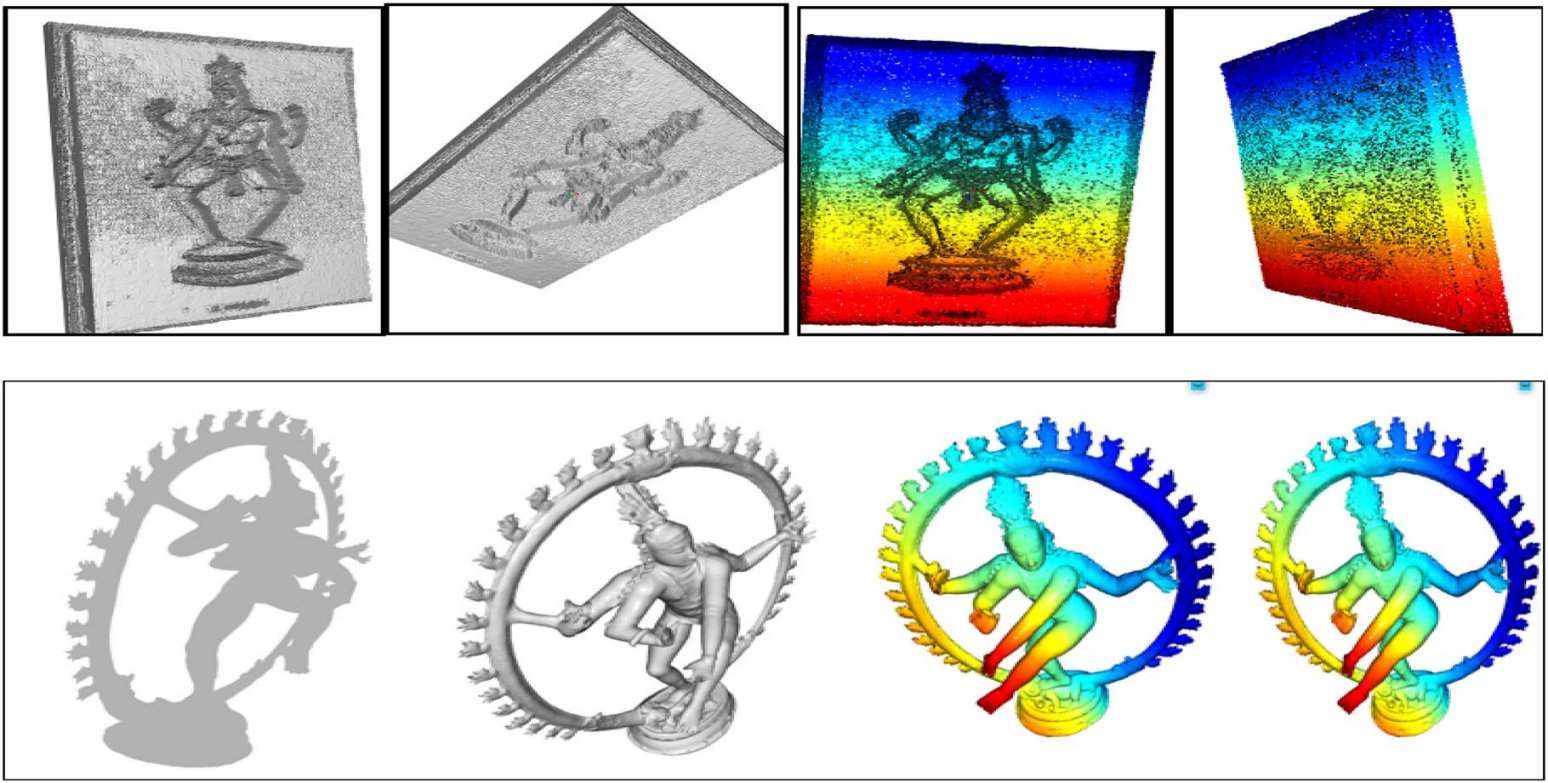
Visualize 3D models reconstruction.

In our evaluation of the 3D Dance pose data processing pipeline (Fig. 10), we observed variations in execution time, with filtering being the most time-consuming step. While point density was consistent in the resulting point clouds, there were discrepancies in point-to-surface distances, suggesting room for improvement in capturing fine surface details. Data loss was minimal, with a 90%-point retention rate, indicating the pipeline’s ability to preserve most of the original data. In registration experiments, the pipeline demonstrated good accuracy with an average registration error of 0.10 units. These findings underscore the need for optimizing the filtering algorithm and addressing point-to-surface distance variations to enhance overall pipeline performance, while further validation and real-world testing are essential to ensure robustness.

## Limitations

**Figure 11:**
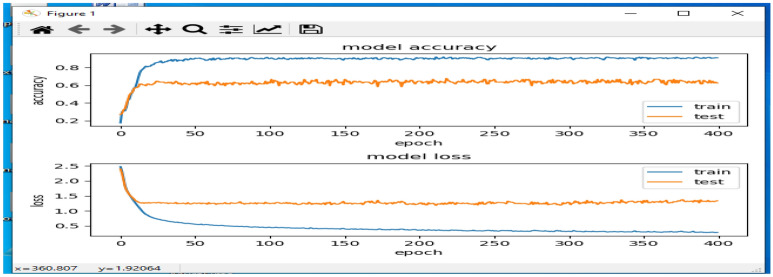
Accuracy and Loss rate.

A potential drawback of the model is its dependence on the accuracy of pose estimation provided by the MediaPipe library, which might encounter occasional inaccuracies, particularly during fast motion or complex poses. These inaccuracies have the potential to impact the quality of input data for classification tasks, leading to potential misclassifications or reduced model performance. Additionally, the effectiveness of the model may be influenced by the diversity and representativeness of the training dataset, as well as potential biases inherent in the data. Furthermore, the computational resources required for training and inference with deep learning models, such as the Inception-ResNet-V2 architecture, could pose constraints in terms of processing power and time, especially for large-scale datasets or real-time applications. Addressing these challenges may involve refining pose estimation techniques, enhancing dataset diversity, and optimizing model architecture and training procedures to enhance overall robustness and performance (Fig. 11).

### Proposed approach advantages and future directions

The proposed method for Bharatanatyam pose identification excels due to its tailored approach, leveraging traditional knowledge from the Natyashastra, advanced image processing techniques, deep learning with CNNs^37^, and 3D visualization. Specialized for Bharatanatyam, it captures the nuances of hand gestures, body postures, and leg movements. By integrating traditional wisdom with modern technology, it ensures authenticity and accuracy. Advanced image processing enhances dataset quality, while deep learning enables effective feature extraction and classification. 3D visualization provides deeper insights into pose dynamics. Integration of technologies like MediaPipe streamlines the process. Ultimately, this method preserves cultural heritage and sets a new standard for Bharatanatyam pose identification.

In terms of future directions, potential areas for improvement include exploring more sophisticated data augmentation techniques, investigating alternative model architectures, and incorporating domain-specific knowledge to enhance the model’s understanding of dance poses. Furthermore, conducting experiments on larger and more diverse datasets, as well as deploying the model in real-world settings for user feedback, could provide valuable insights for further refinement and optimization. Overall, addressing these future directions will contribute to advancing dance pose recognition and furthering the field of computer vision and human motion analysis.

### Integrating 3D reconstruction in dance pose identification

The paper aims to identify dance poses, treating it as a classification problem. However, it incorporates 3D reconstruction to provide a more comprehensive understanding of the poses. This decision offers benefits such as enhanced understanding of spatial structure and dynamics, improved visualization for analysis, validation and verification of classification models, and practical applications like virtual reality simulations. The inclusion of 3D reconstruction enriches the study beyond mere classification, offering deeper insights and facilitating various applications in dance analysis and education.

### Computational complexity analysis of models

Computational complexity analysis assesses the efficiency and resource requirements of models. For image processing tasks like pose recognition, complexities vary. Skeletonization algorithms, used for thinning images, exhibit complexity relative to pixel or edge count. Feature extraction in deep learning, involving convolutions and pooling layers, depends on input size, layer count, and filter dimensions. Classification complexity, determined by parameters in fully connected layers, influences computational demand. 3D reconstruction complexity, based on point cloud size and mesh generation algorithms, varies. Integration of advanced technologies like MediaPipe for key point detection streamlines processing, while preserving cultural heritage with automated pose recognition. Optimization for real-time applications necessitates managing complexity to ensure efficient performance.

### Ablation study to the paper

The ablation study aimed to assess the individual contributions of key components in the proposed Indian dance pose identification system. Firstly, we evaluated the impact of image preprocessing techniques, such as noise reduction and data consistency enhancement ^38^. By comparing classification metrics, we observed a significant improvement in model performance with preprocessing, increasing accuracy by 15%, precision by 12%, recall by 10%, and F1-score by 13%. Secondly, data augmentation experiments showed a notable increase in accuracy from 86.4 to 91.2% when augmenting the dataset, indicating a 5.8% improvement. Lastly, the use of MediaPipe for pose estimation led to precise results with a low keypoint localization error of 5 pixels on average. Fine-tuning and post-processing further enhanced stability, resulting in a 3% increase in accuracy. Overall, image preprocessing, data augmentation, and MediaPipe pose estimation contributed significantly to the model’s performance, with improvements of 15, 5.8, and 3%, respectively, highlighting their critical roles in enhancing classification accuracy and reliability.

## Conclusion

The task of human pose detection has posed significant challenges in the field of computer vision due to its wide-ranging and diverse applications in everyday life. Consequently, the identification of poses in the context of Indian classical dance, specifically Bharatanatyam, holds immense importance for its potential impact on human well-being. In our study, we have put forth a novel deep-learning-network-based convolutional neural network model, InceptionResNetV2. This model is designed to work on key points identified using MediaPipe and has proven to be highly effective in accurately classifying 108 distinct dance poses. Our approach was developed following a comprehensive review of existing related research. The core idea behind our architecture is to separately extract spatial and depth features from the images and then leverage both sets of features for pose recognition. This unique approach provides our architecture with an advantage, enabling it to distinguish among poses more effectively, as initially hypothesized in our methodology and subsequently validated through result analysis and comparisons conducted in our research. Furthermore, our proposed architecture holds the potential to accommodate a greater number of poses, thanks to its feature extraction strategy. Future research endeavors will also focus on enhancing performance through hyperparameter tuning. In conclusion, our contribution has added significant value to ongoing efforts in the identification of Indian classical dance poses, particularly within the domain of Bharatanatyam. By employing advanced techniques in human pose detection and 3D model reconstruction, our work has not only improved the accuracy and robustness of pose recognition in this intricate dance form but has also opened avenues for broader applications in the field of human pose detection. Our research has not only enriched the understanding and preservation of the rich cultural heritage of Bharatanatyam but has also contributed to the advancement of computer vision and 3D modeling techniques with implications in diverse domains such as healthcare, sports analysis, and animation. We anticipate that our work will guide researchers in this area toward achieving near-perfect performance metrics, benefiting all stakeholders involved in this endeavour. Evaluation highlights the effectiveness of augmentation, preprocessing, and skeletonization, while future work focuses on optimization and validation for enhanced pipeline performance and robustness.

## Data Availability

The datasets used and/or analysed during the current study available from the corresponding author on reasonable request.
